# Exploring the lived experiences of women with breast cancer: a qualitative study across different stages of the disease

**DOI:** 10.3389/fpubh.2026.1872393

**Published:** 2026-07-01

**Authors:** Abdullah Almaqhawi, Suha Albahrani, Jamela Abdullah Turkistani, Abdullah Almulhim

**Affiliations:** Department of Family and Community Medicine, College of Medicine, King Faisal University, Al Ahsa, Saudi Arabia

**Keywords:** breast cancer, lived experience, psychosocial support, qualitative study, survivorship

## Abstract

**Background:**

Breast cancer is the most commonly diagnosed malignancy among women in Saudi Arabia and is frequently diagnosed at younger ages and more advanced stages than in many high-income settings. Qualitative accounts of how Saudi women experience breast cancer across the disease trajectory remain scarce. To explore the diagnosis experience, treatment journey, psychological impact, and social and healthcare support gaps of women across different stages of breast cancer attending a charitable cancer support organization in Al-Ahsa, Saudi Arabia.

**Methods:**

This qualitative study was conducted using semi-structured, in-depth interviews with 12 women diagnosed with breast cancer at various stages. Participants were recruited through a charitable cancer support organization in Al-Ahsa, Saudi Arabia. Interviews were conducted face-to-face in Arabic, audio-recorded, verbatim, and translated into English. Data were analyzed using reflexive thematic analysis to identify shared patterns and divergent experiences.

**Results:**

A total of five themes were identified. Diagnosis experience was reported as profound emotional disruption described mainly as shock, fear and disbelief which were further intensified by delays in diagnosis and referral pathways. Treatment journeys were multimodal and patients mainly emphasized the difficulties faced in symptoms management and the lack of preparedness for the physical and emotional burden of the therapeutic modalities. Psychological impact ranged from fear to severe distress, some participants reported emotional coping by social support, and faith-based acceptance as the main protective factors while others highlighted the role of marital struggles and fear for the future in exacerbating their stress.

**Conclusion:**

Within this single-site charitable-organization sample, women’s experiences were shaped by timeliness of diagnosis, preparedness for treatment, symptom control, quality of clinician communication, marital stability, and faith-based meaning-making. Findings are exploratory and context-specific; they highlight potential leverage points, earlier referral, structured pre-treatment education, proactive psychosocial screening, and sensitivity to marital and cultural context, that warrant evaluation in larger, multi-site Saudi studies.

## 1 Introduction

Breast cancer is the most prevalent form of cancer in women worldwide, and it is a significant public health issue due to its prevalence, the prolonged recovery process, and the significant impact on mental health. The Global Cancer Observatory (GLOBOCAN) indicates that breast cancer accounts for approximately 24–25% of all cancer diagnoses among women worldwide, with a growing prevalence observed in a variety of geographic and socioeconomic contexts (1, 2). Improvements in screening, early diagnosis, and multimodal treatment have greatly increased survival rates, especially in countries with high and middle income (3). Consequently, a growing number of women are surviving longer with breast cancer, highlighting the importance of quality of life and survivorship issues beyond conventional clinical outcomes.

Historically, breast cancer research has focused primarily on biomedical endpoints such as tumor biology, treatment efficacy, recurrence, and mortality. While these outcomes remain critical, they provide an incomplete picture of the burden of disease. A growing body of literature highlights that the diagnosis and treatment of breast cancer are often accompanied by profound psychological and emotional distress, including anxiety, depression, fear of recurrence, and uncertainty regarding the future (4, 5). These emotional challenges may persist long after the completion of active treatment, affecting women’s overall well-being and daily functioning.

In addition to psychological distress, women with breast cancer frequently experience significant physical and social challenges. Treatment-related side effects such as fatigue, pain, lymphedema, and menopausal symptoms can interfere with personal independence and social participation (6). Changes in body image and sexuality, particularly following mastectomy or chemotherapy-induced alopecia, may negatively impact self-esteem and intimate relationships (7). Social roles within the family and workplace are also commonly disrupted, with many women reporting difficulties balancing treatment demands with caregiving responsibilities, employment, and social expectations (8).

The lived experience of breast cancer is not homogeneous and varies across the disease continuum. Women diagnosed with early-stage breast cancer often describe intense emotional distress at the time of diagnosis, followed by ongoing fear of recurrence during the survivorship phase (9). In contrast, women living with advanced or metastatic breast cancer face unique challenges related to chronic treatment, symptom burden, and awareness of limited life expectancy, often accompanied by heightened existential distress and concerns about autonomy and future planning (10). These stage-specific differences underscore the need for research that explicitly examines experiences across all stages of breast cancer.

Age and cultural context further shape women’s experiences of breast cancer. Younger women may struggle with fertility preservation, premature menopause, body image concerns, and the impact of illness on career development and parenting (11). Older women may experience compounded challenges related to comorbidities, functional decline, and social isolation. Cultural beliefs, stigma surrounding cancer, family dynamics, and health literacy influence how women interpret their diagnosis, seek support, and engage with healthcare services (12). Understanding these contextual factors is essential for delivering culturally sensitive and patient-centered care.

Qualitative research methods have proven particularly valuable in capturing the complexity of breast cancer experiences. Through in-depth interviews and narrative analysis, qualitative studies have illuminated women’s perspectives on diagnosis disclosure, treatment decision-making, coping strategies, and interactions with healthcare providers (13). Such approaches allow for a deeper exploration of meanings, emotions, and unmet needs that are often overlooked in quantitative research.

Despite existing qualitative studies, there remains a gap in research that comprehensively explores the lived experiences of women across all breast cancer stages while simultaneously considering age, cultural background, and treatment modality. Addressing this gap is crucial for informing holistic, stage-appropriate psycho-oncological interventions and support services. By exploring women’s narratives from diagnosis through treatment and survivorship, this study aims to contribute meaningful insights that can enhance patient-centered care, guide supportive services, and inform public health strategies for women living with breast cancer (14).

In Saudi Arabia, breast cancer is the most commonly diagnosed malignancy among women and accounts for a disproportionate share of cancer-related morbidity and mortality in this population. National and regional series have consistently reported that Saudi women present at younger ages and at more advanced stages than women in many high-income countries, with a substantial proportion of patients diagnosed before the age of 50 (15–17). Care is delivered primarily through state-funded tertiary oncology centres, and recent national investments in early-detection programmes (e.g., the Sehhaty platform and school-based awareness campaigns) have begun to shift the diagnostic pathway. Alongside this, a growing network of charitable cancer support organisations, such as the Tafaoul Charitable Society for Cancer Control in Al-Ahsa, provides psychosocial, educational, and practical support for patients and their families, often filling gaps that sit outside formal clinical services. Within this specific sociocultural context, in which faith, marital and family structure (including polygamous marriage), preferences for same-gender clinical care, and stigma around illness disclosure meaningfully shape experience, qualitative accounts of how women actually move through the stages of breast cancer remain scarce. The present study addresses that gap by focusing on women engaged with a charitable cancer support organisation in Al-Ahsa and by explicitly attending to stage-specific differences across the disease trajectory.

## 2 Methods

### 2.1 Research design

This study used a qualitative design with semi-structured, in-depth interviews in Arabic to explore lived experiences of women diagnosed with breast cancer across different stages and treatment trajectories during October 2025 to November 2025 as well as interpret the phenomenon by examining common experiences and shared viewpoints (18). Data were collected through semi-structured interviews to obtain rich qualitative insights into participants’ experiences, attitudes, and beliefs (19). Purposive sampling was used to recruit patients who registered in the database at Charitable Cancer Control Society in Al-Ahsa (Tafaoul), Al-Ahsa, Saudi Arabia. The Tafaoul Charitable Society for Cancer Control in Al-Ahsa, Saudi Arabia, a non-profit organization that provides comprehensive support services for patients with cancer, including psychosocial support, health education, and community-based care. The organization serves as a key community partner in cancer prevention, patient support, and awareness initiatives within the Al-Ahsa region. Participant recruitment was conducted in collaboration with the Tafaoul Charitable Society for Cancer Control in Al-Ahsa, Saudi Arabia. Recruitment messages were disseminated via telephone using the society’s database, and study invitations were also distributed in poster format during support group meetings. Women aged 18 years and older with a diagnosis of breast cancer at any stage were considered eligible for participation.

Invitations were sent to more than 300 women, of whom approximately 20 expressed interest in participating. All respondents met the eligibility criteria. Prior to conducting the interviews, informed consent was obtained from all participants. A total of 12 interviews were completed. Data collection ended when the research team judged that the interviews carried sufficient information power for the study’s focused aims, given the narrow research question, the dense and stage-diverse sample, and the quality of the interview dialogue; this framing is used in preference to thematic “saturation,” which is contested within reflexive thematic analysis (20). Participants varied by age, family context (married, divorced, single; presence/age of children), and clinical trajectory (e.g., early-stage post-treatment survivorship, ongoing endocrine therapy, recurrence concerns, and metastatic disease). Each participant was assigned a sequential pseudonymous code (Participant 1 to 12), direct identifiers were removed, and potentially identifying combinations of attributes (for example, exact age together with stage, family structure, and sensitive events) were minimised in the reported quotations and tables to protect confidentiality. No individual participant can be re-identified.

The interview guide was developed following a comprehensive review of peer-reviewed qualitative and psychosocial oncology literature addressing breast cancer diagnosis, treatment decision-making, psychosocial impact, social support, quality of life, and survivorship. All interview domains were grounded in established evidence to ensure content validity and methodological rigor (21–28). The guide was pilot-tested with two Arabic-speaking individuals to assess clarity, cultural appropriateness, and acceptability. Feedback from the pilot testing informed refinements to the interview questions. The interview transcripts in Arabic were translated into English by AA and double-checked with the research team authors to ensure translation validity. Coding was performed on the original Arabic transcripts, and only the quotations selected for reporting were translated into English. Back-translation of the quotations was also carried out by AA.

The interviews explored participants’ experiences across multiple domains, including the diagnostic journey, information provision, treatment decision-making, physical side effects, psychological well-being and coping strategies, family and community relationships, healthcare experiences, financial and occupational impacts, survivorship concerns, and culturally embedded understandings of illness (see Appendix 1 for the interview guide).

Two pilot interviews were conducted to ensure comprehension, neutrality, and appropriateness of the questions, and the interview guide was revised accordingly prior to the main data collection (18). Face-to-face semi-structured interviews were conducted in Arabic, each lasting between 25 and 35 min (mean ≈ 30 min). Although relatively brief, the interviews followed a focused interview guide and continued until no new themes emerged, which accounts for the depth of the resulting data. and were audio-recorded in a private room within the Charitable Cancer Control Society in Al-Ahsa (Tafaoul). Data were analysed using reflexive thematic analysis (29). Coding was performed on the Arabic-language transcripts by the primary researcher; the senior author independently reviewed the coding and the developing themes, and divergences were resolved through reflexive discussion to deepen interpretation rather than as an inter-coder reliability exercise, consistent with the assumptions of reflexive thematic analysis (29, 30). Discrepancies were resolved through discussion, and the study adhered to the COREQ reporting guidelines (31). Participation was optional, and informed consent was obtained from all participants prior to data collection. Confidentiality and anonymity of participants were maintained throughout the study, with all data securely retained and used exclusively for research purposes. Interview audio files and transcripts were managed using Microsoft Word and Microsoft Excel for organisation, coding, and retrieval; no dedicated qualitative data analysis software (e.g., NVivo, MAXQDA) was used. No repeat interviews were carried out. Transcripts were not returned to participants for verification, as many participants preferred limited additional contact after the interview and to minimise the emotional burden of revisiting interview content; credibility was instead supported through in-interview paraphrasing and member-checking of key statements in real time, and independent review of the coding and the developing themes by the senior author; inter-coder agreement was not treated as a quality criterion, in keeping with reflexive thematic analysis. To manage the emotional risks of these sensitive interviews, the interviewer was able to pause or stop the interview on signs of acute distress, and any participant disclosing acute risk during the interview (including the participant who reported a suicide attempt) was referred to the treating oncology team and/or mental-health services and offered a debrief.

### 2.2 Data analysis

A reflexive thematic analysis approach was used. Transcripts were read repeatedly to achieve familiarization, then coded inductively for meaning units relevant to the research objectives. Codes were iteratively clustered into candidate themes, which were refined by checking coherence within themes and distinction between themes. Attention was paid to convergence (shared patterns across participants), divergence (stage-, age-, and context-specific differences), and “negative cases” that complicated or contradicted dominant narratives. English quotations below are translations, selected for their representativeness and conceptual clarity.

## 3 Results

### 3.1 Participant characteristics

The twelve participants ranged in age from 37 to 60 years at the time of interview (median approximately 40) and from 33 to 46 years at the time of diagnosis (median approximately 39). Years since diagnosis ranged from less than one year to approximately 18 years, representing the full survivorship trajectory from early post-treatment to long-term survivorship. The sample included women with early-stage disease (Stages I–II), locally advanced disease (Stage III), and metastatic disease (Stage IV), as well as women with recurrence. Family situations and employment contexts varied widely, shaping needs and vulnerabilities. Table 1 shows participants’ characteristics. Table 2 summarizes the participant-by-code matrix (presence/absence). Through the process of conducting a thematic analysis, a total of five refined themes and 30 sub-themes were discovered (Table 3).

**Table 1:** Characteristics of the qualitative interview sample.

Participant	Age	Marital/family context	Year of diagnosis	Stage/grade as stated	Treatment trajectory described
1	41 now; 40 at diagnosis	Married, 4 children; employment loss then association-supported training	~1 year prior	“Between stage 2 and 3”	Detected via screening campaign; reconstruction insisted on
2	40 now; 33 at diagnosis	Single; type 1 diabetes	2017	Stage 3	Chemo, mastectomy + nodes, radiotherapy, endocrine therapy 5–6 yrs
3	60 now; ~42 at diagnosis	Married; two adult children; works in an association	2007	Stage 2	Chemo → mastectomy → radiotherapy
4	37 now; 35 at diagnosis	Pharmacist; mother of two children (12 and 4)	2022	Stage 2	Delayed escalation after reassurance; biopsy later confirmed malignancy
5	45 now; 39 at diagnosis	Single; previously taught Qur’an/Tajweed	2020	Stage 2	“Possibly no chemo” initially but anticipated need
6	39 now; ~38 at diagnosis	Married; active lifestyle	2025	Stage 2; HR+	Chemo → surgery → radiotherapy → endocrine therapy
7	39 now; 36 at diagnosis	Mother of two	2022	Stage 1 progressing to Stage 2 after referral delay	Mastectomy, chemo, nodes surgery, radiotherapy, reconstruction, endocrine therapy
8	48 now; 38–39 at diagnosis	Married; son and daughter	~10 years prior	Stage not recalled	Axillary + partial breast surgery, chemo, radiotherapy, endocrine therapy
9	52 now; 43 at diagnosis	Married; son and daughter	2016	Stage 2	Chemo → surgery → radiotherapy; later lymph node recurrence and weekly chemo for a year
10	48 now; ~46 at diagnosis	Mother of four children	~2 years prior	Stage 4	Chemo + nuclear therapy + biologics + surgery + radiotherapy
11	40 now; 39 at diagnosis	Divorced; two young children; financial instability; diabetes	~1 year prior	Stage 4	Biologic + endocrine therapy + anticoagulation for pulmonary embolism
12	40 now; 34 at diagnosis	Teacher; treated in Riyadh; living in Al-Ahsa	2019	Histological grade 3; stage not reported	Two surgeries, 25 radiotherapy sessions; reconstruction 2024; new contralateral lump 2025 awaiting result

**Table 2:** Participant-by-theme matrix: distribution of codes across the sample.

Participant	DSH	DDL	IDC	MTB	MET	PDS	SUI	FAI	MAR	SOC	NAV	CUL	SUR
Participant 1	✓	✓		✓				✓		✓			✓
Participant 2	✓	✓		✓		✓		✓		✓	✓		✓
Participant 3	✓	✓		✓		✓		✓		✓	✓		✓
Participant 4	✓	✓		✓		✓		✓		✓	✓		✓
Participant 5	✓	✓		✓		✓		✓	✓	✓	✓		
Participant 6	✓	✓	✓	✓		✓		✓		✓			
Participant 7	✓	✓		✓		✓		✓		✓	✓	✓	✓
Participant 8	✓	✓		✓		✓		✓		✓			✓
Participant 9	✓	✓		✓						✓	✓	✓	✓
Participant 10	✓	✓		✓	✓	✓	✓		✓	✓	✓	✓	✓
Participant 11	✓	✓	✓	✓	✓	✓		✓	✓	✓	✓		✓
Participant 12	✓	✓		✓		✓		✓	✓	✓	✓		✓

Codes shown: DSH [diagnosis shock/fear], DDL [delay in seeking/diagnostic pathway barriers], IDC [information/disclosure problems], MTB [multi-modality/long treatment burden], MET [metastatic constraints], PDS [psychological distress], SUI [suicidality], FAI [faith/meaning-making], MAR [marital strain/rupture], SOC [social support outside immediate family], NAV [navigation/coordination difficulties], CUL [cultural/gendered care preferences], SUR [surveillance/follow-up & future planning].

**Table 3:** Emergent themes and subthemes from participant interviews.

Number	Theme	Subtheme	Illustrative quotation (English translation)	Participant evidence
1	Diagnosis experience: shock, uncertainty, and the cost of delays	Shock and disbelief at diagnosis	“It was a shock… something I could never forget… I did not expect it even 1%.”	Participant 8
		Early reassurance and delayed confirmation	“He told me it’s a benign lump… no need for any procedure… then it grew… and the biopsy showed it was malignant.”	Participant 4
		Referral delay and stage progression	“It was confirmed as stage one… but the referral was delayed four months… which led to stage two.”	Participant 7
		Awareness and screening as an enabling pathway	“I discovered it through early detection… messages from Sehhaty and awareness campaigns at my children’s schools.”	Participant 1
		Awareness-driven self-examination prompting referral	“I found a lump during awareness campaigns… I went for early detection and then they did imaging and referred me.”	Participant 2
2	Treatment journey: intensity, body disruption, and uneven preparation for side effects	Multi-modality and prolonged treatment trajectories	“They decided on chemotherapy, then mastectomy, then radiotherapy… and later I needed weekly chemotherapy for a full year.”	Participant 9
		Sequenced pathway with long endocrine therapy	“Chemotherapy… then mastectomy with lymph node removal… then radiotherapy… then endocrine therapy for five to six years.”	Participant 2
		Being well-prepared by clinicians reduces fear	“They gave me adequate and sufficient explanation at every step… I first refused mastectomy… then accepted when they explained it protects from spread.”	Participant 2
		Unmet needs: inadequate information, pain control, and early coordination	“Very bad… because of lack of information about side effects, severe pain without enough painkillers early on, and difficulty communicating with coordinators.”	Participant 10
		Side effects as identity-threatening bodily change [hair loss/surgery loss]	“I was sad about losing my hair during chemotherapy… and I grieved losing an important part of my body.”	Participant 2
		Endocrine therapy effects and reconstruction barriers [weight gain]	“The most troubling side effect was weight gain… it became a barrier even to reconstruction.”	Participant 2
		Chemotherapy as the hardest phase physically and psychologically	“Chemotherapy was the hardest… exhaustion, headaches, dizziness, balance problems… thoughts that I might die and leave my children.”	Participant 8
3	Psychological impact: fear, meaning-making, and the extreme edge of distress	Fear of death and anticipatory grief for children	“Sometimes I thought I might die… how will I leave my children, and how will they live?”	Participant 8
		Children’s future as the heaviest emotional burden	“The biggest thing I carried was my daughters… my daughters’ future.”	Participant 4
		Faith-based acceptance and emotional steadiness	“I did not collapse… I leave my affairs to God’s decree.”	Participant 6
		Religious principle guiding daily coping	“I follow the verse ‘God does not burden a soul beyond its capacity’… I managed the home within my capacity.”	Participant 1
		Extreme distress and suicidality as intersection of unmet needs	“I tried suicide three times… due to lack of information about side effects, uncontrolled pain early on, my husband abandoning me, fear for my children…”	Participant 10
4	Social support and relationships: family as buffer, stigma as wound, and marital instability as a risk amplifier	Family support as the main protective buffer	“My family were the biggest supporters.”	Participant 8
		Workplace stigma and appearance-based harm	“A small number spoke negatively about my appearance and my hair… so I avoided them.”	Participant 8
		Protecting privacy and avoiding distressing looks	“I avoided going out so relatives who did not know would not see my changed appearance and give distressing looks.”	Participant 12
		Pity looks leading to withdrawal	“People looked at me with pity… it bothered me… I tried to isolate myself.”	Participant 7
		Marital abandonment and social exposure as compounded trauma	“My husband’s abandonment… forced me to face a whole society… pity looks and improper treatment.”	Participant 10
		Marital support as protection even in complex family structure	“My husband supported me… even though he had a second wife.”	Participant 9
5	Healthcare system experience: respectful care exists, but coordination and information gaps can be consequential	Respectful care alongside isolated harmful encounters	“Most were kind, but one doctor raised his voice… many people complained about him.”	Participant 8
		Coordination problems: delayed referral	“Referral was delayed four months…”	Participant 7
		Coordination problems: crowded spaces and waiting	“Examination rooms were crowded… and appointment waiting time was long.”	Participant 12
		Coordination problems: difficulty contacting coordinators early in chemo	“Communication with coordinators was very difficult.”	Participant 10
		Information opacity: discovering stage by chance	“I found out I was stage four by chance when I asked for a report for charity support.”	Participant 11
		Psychological care inconsistently offered and variably needed	“Psychological support wasn’t offered, but I did not feel I needed it.”	Participant 9
		Need for proactive psycho-oncology contact	“No one from mental health contacted us directly… we needed someone to talk to.”	Participant 12

### 3.2 Diagnosis experience: shock, uncertainty, and the cost of delays

Diagnosis was commonly described as an emotional rupture. Even when participants perceived themselves as “prepared,” the news carried fear and destabilization. Participant 8 described diagnosis as “a shock… something I could never forget” and emphasized how unlikely she felt it was (“I didn’t expect it even 1%”). Participant 4 similarly framed the moment as a major shock, intensified by family history and responsibility for children’s futures.

A recurring pattern was delay or misdirection in the pathway to diagnosis. Participant 4 described early reassurance that the mass was benign and required no further action, followed by later growth and subsequent biopsy confirmation. Participant 7 explicitly linked referral delay to stage progression: she was initially told it was Stage I, but a four-month transfer delay preceded progression to Stage II and a more intensive treatment plan. By contrast, some women were diagnosed through structured screening and awareness channels. Participant 1 described detection through an early screening campaign promoted via health-platform messages and school-based awareness campaigns. Participant 2 similarly noted that she detected a lump through self-examination during awareness campaigns, triggering formal imaging and referral.

These accounts suggest that awareness pathways can shorten time-to-diagnosis, while system delays and initial reassurance can lengthen it.

### 3.3 Treatment journey: intensity, body disruption, and uneven preparation for side effects

Treatment trajectories were multi-modal and often prolonged. Participants described chemotherapy, surgery (partial or mastectomy), radiotherapy, and endocrine therapy, with some also describing biological or nuclear treatments. Participant 2 described a clearly sequenced plan: chemotherapy, mastectomy with lymph node removal, radiotherapy, then long-term endocrine therapy.

Participant 6 described chemotherapy (16 sessions) followed by surgery, radiotherapy, and endocrine therapy for HR + disease. Participant 8 reported combined axillary surgery, partial breast surgery, chemotherapy, radiotherapy, and endocrine therapy. What separated experiences was not only the class of treatment received, but how prepared participants felt. Participant 2 reported that she was given “adequate and sufficient explanation at every step,” even while initially resisting mastectomy because of bodily “disfigurement,” and then accepting after it was framed as protective against spread.

In other cases, lack of preparation and symptom management contributed to severe distress. Participant 10 described profound suffering during early treatment, explicitly linking her crisis to insufficient information, severe pain without adequate analgesia, and difficulty communicating with coordinators early on. Side effects were discussed as both physical and identity-threatening. Participant 2 spoke of hair loss sadness during chemotherapy and grief over losing “an important part of my body” during surgery.

She later described weight gain as the most troubling endocrine side effect because it became a barrier even to reconstruction. Participant 8 described chemotherapy as the hardest phase psychologically and physically, with exhaustion, headaches, dizziness, balance issues, and intrusive thoughts about death and her children’s future.

### 3.4 Psychological impact: fear, meaning-making, and the extreme edge of distress

Many women reported fear, anxiety, and psychological strain, often centered on mortality and children. Participant 8’s account showed anticipatory grief and fear of leaving children. Participant 4 described her heaviest burden as her daughters and their future, even more than treatment. Some participants framed emotional coping through faith-based acceptance. Participant 6 described fear and worry without “collapse,” attributing steadiness to being mentally prepared and to religious surrender (“I leave my affairs to God’s decree”).

Participant 1 described applying a religious principle of “God does not burden a soul beyond its capacity” as a rule for managing household demands during treatment. At the most severe end, Participant 10 reported suicide attempts during treatment, tying this to inadequate side-effect education, uncontrolled pain early in treatment, marital abandonment, fear for her children, and the impact of altered appearance on her young son. Her narrative is a clear example of how psychological crisis can emerge from the intersection of physical suffering, informational gaps, and social rupture, rather than “depression” as an isolated symptom.

### 3.5 Social support and relationships: family as buffer, stigma as wound, and marital instability as a risk amplifier

Family support often functioned as a stabilizing buffer. Participant 8 described family as “the biggest supporter,” and noted that although most colleagues were fine after her year-long absence, a small number were harmful through negative comments about appearance and hair, prompting avoidance.

This illustrates how workplace interactions can quietly produce stigma and self-isolation even when family support is strong. Some participants protected their privacy to avoid social reactions. Participant 12 described avoiding going out partly to prevent relatives who did not know about her illness from seeing her changed appearance and giving her distressing looks.

Participant 7 similarly described withdrawing from people who looked at her with pity. Marital context emerged as a major axis of vulnerability. Participant 10 described profound harm from her husband’s treatment and abandonment, while also describing the experience as forcing her to “face a whole society,” including pitying looks and “improper” treatment. Conversely, Participant 9 described her husband as a key supporter, even noting a complex family structure [husband had a second wife], but experiencing this as positive support rather than relational injury. These divergent accounts suggest marital dynamics can shift from protective to destructive, strongly shaping emotional safety during treatment.

### 3.6 Healthcare system experience: respectful care exists, but coordination and information gaps can be consequential

Women reported both excellent and harmful encounters. Participant 8 described generally kind care but identified “one doctor” who was verbally harsh (“raised his voice”), later learning many others complained about him. Coordination problems appeared in multiple forms: delayed referral (Participant 7), crowded and unpleasant diagnostic spaces and appointment waiting (Participant 12), difficulty contacting coordinators early in chemotherapy (Participant 10), and a striking example of informational opacity where a patient discovered she was Stage IV “by chance” when requesting a report for charity support (Participant 11).

Psychological care was inconsistently offered. Participant 9 said psychological support was not offered, but she did not feel she needed it. Participant 12 argued that psychological support is important and noted that no one from mental health contacted them directly during periods of high need.

### 3.7 Stage-stratified experiences across the disease trajectory

Although the five cross-cutting themes applied to the sample as a whole, the narratives of women at different stages differed in qualitatively meaningful ways. At the early-stage end of the trajectory (Stages I–II, e.g., Participants 1, 4, 6, 7), the dominant emotional texture combined the shock of diagnosis with preoccupations about treatment tolerability, body image, and the risk of recurrence. Several of these women described a “system encounter” narrative in which delay, early reassurance, or slow referral played a defining role (Participants 4 and 7), and in which awareness channels and structured screening (Participants 1 and 2) were experienced as protective. Children’s welfare featured prominently as the emotional anchor of distress but was framed in terms of temporary disruption rather than anticipated loss.

Locally advanced disease (Stage III, e.g., Participant 2) introduced a qualitatively denser treatment burden: longer multi-modal regimens, staged reconstruction decisions, endocrine therapy for 5–6 years, and lingering body-image and weight-related concerns that extended long after active treatment. Women at this stage most often described the clinician-communication axis as the pivot between endurable and overwhelming experience: well-prepared patients described being able to accept feared interventions (Participant 2), while poorly prepared patients described acute crises during chemotherapy (Participant 10).

Women with metastatic disease (Stage IV, Participants 10 and 11) and those with recurrence (Participant 9) described a different constellation. Here distress was shaped less by discrete treatment events and more by the chronicity of treatment, existential uncertainty, and the awareness that life-plans had to be reorganised around an open-ended prognosis. Participant 10’s account of suicidal crisis during early metastatic treatment illustrated how informational failures, uncontrolled pain, marital abandonment, and fear for her children compounded into a safety event rather than a discrete mood disorder. Participant 11’s discovery of her Stage IV status “by chance,” while requesting a report for charity support, revealed a distinctive informational rupture specific to advanced disease. Participant 9’s recurrence narrative, lymph-node recurrence requiring a year of weekly chemotherapy, emphasised the way surveillance findings re-open, rather than resolve, the psychological work of the survivorship phase.

Long-term survivors (e.g., Participants 3, 8, and 12, with 6–18 years since diagnosis) described a third register: a fluctuating baseline of surveillance-related vigilance, sporadic reactivations of distress at the time of investigations or new lumps (Participant 12’s 2025 contralateral lump), and a slow reconfiguration of identity around “life after treatment” rather than “life with cancer.” The stage-specific patterns outlined here are offered descriptively, given the qualitative and exploratory nature of the dataset; they are not intended as a formal typology but as an analytic lens that distinguishes what is shared across the trajectory from what is specific to each stage.

## 4 Discussion

Participants described breast cancer diagnosis as an emotionally rupturing moment marked by shock, fear, and uncertainty, often intensified when diagnosis pathways involved reassurance, delay, or fragmented referral processes. Treatment journeys were typically long and multi-modal, with distress shaped not only by physical side effects but also by disruption to identity, body image, and daily roles. Psychological responses ranged from worry and anticipatory grief, especially concerning children’s futures, to severe crisis when pain, poor preparation, and social rupture intersected. Social support, particularly from family, buffered distress, while stigma, pitying reactions, workplace discomfort, and marital instability amplified vulnerability. Healthcare experiences were mixed: respectful and supportive care existed, but coordination failures, communication gaps, and limited access to proactive psychosocial support sometimes had serious consequences.

Awareness and screening are central to modern survivorship, as regular surveillance for recurrence and new primary cancers allows for earlier detection and faster diagnosis. While routine laboratory tests for asymptomatic patients are not supported by current data, a detailed cancer-related history and physical examination are critical to reducing the uncertainty inherent in the “low-risk for relapse” state that survivors inhabit (32). The burden of treatment extends far beyond clinical symptoms, deeply affecting a woman’s identity and social roles. The physical losses, including breasts, hair, and fertility, often disrupt a woman’s sense of femininity and self-image (33). This identity-related burden is particularly acute for young survivors, who may struggle to balance recovery with their roles as mothers, wives, or workers. Many women strive to achieve a “new normal” where they can fulfill everyday activities without the illness serving as a constant hindrance to their identity (34).

Psychological breakdown in survivors is rarely a result of mental health symptoms in isolation; instead, it is linked to a cluster of pressures. These include chronic pain, fatigue, a perceived lack of information or “mismatch” between medical explanations and lived reality, and the stigma of the disease. The phenomenon of “chemobrain,” which includes deficits in concentration and memory, often comes as a surprise and can lead to significant distress and anxiety if not acknowledged as a valid side effect by clinicians (7, 35, 36).

The health system plays a dual role in both mitigating and exacerbating survivor distress. Delays in the transition from oncology to primary care, combined with referral barriers and poor coordination, increase the psychological burden on patients. The waiting environment and the period of anticipating test results are often described as the most agonizing forms of existence, where the “future” disappears into the results (37).

Communication quality significantly shapes trust and treatment acceptance. A “mismatch” often exists where clinicians focus on measurable symptoms while patients struggle to explain their subjective reality. When clinicians fail to explore a patient’s individual concept of “normality, “it can lead to inappropriate advice and unrealistic expectations. Conversely, clear explanations and validation of symptoms like chronic fatigue or cognitive impairment build the trust necessary for survivors to adhere to long-term endocrine therapies (38–40).

A major shortcoming in the current system is the lack of proactive psychological follow-up. Many women are discharged back to primary care without a survivorship care plan or a clear roadmap for support services. This leaves them unsupported during the peak vulnerability of the post-treatment phase, often described as a “reorientation” period where they must reconcile with the loss of their old existence (32).

Family support is consistently identified as the primary protective factor and a form of “social capital” essential for coping. Spouses, partners, and parents often provide the consistent, continuing care necessary for the survivor to manage long-term side effects. For some, this support includes spirituality, where faith in a supreme being provides hope, companionship, and a framework for making meaning of the illness (41).

Conversely, stigma and the fear of being “othered” can push women toward withdrawal and isolation. This is particularly true for cancers with lower “prestige” or higher taboos, such as gynaecological cancers, where patients may disguise their cancer site to avoid social embarrassment. Appearance changes, such as baldness or mastectomy scars, can lead women to comply with societal rules to “not look ill” by using wigs or prostheses, even when they find them uncomfortable (42).

Marital instability or a partner’s inability to understand the survivor’s feelings represents a major risk factor for safety and wellbeing. Some women report that the medical discourse pressures them to seek reconstructive surgery “for their husband,” which can lead to conflict if the woman’s own priorities are ignored. While many partners are supportive, the emotional strain of the diagnosis can lead to instances where a partner is unable to cope, resulting in abandonment during a time of extreme physical and psychological vulnerability (43). Consistent with these accounts, recent Saudi evidence documents that intimate partner violence and psychological abuse are reported by a substantial minority of women with breast cancer, with verbal, emotional, and controlling behaviours the most common forms and spousal unemployment and larger family size among the associated factors; this reinforces the need to screen for relational safety rather than assume that marriage is protective (44, 45).

These accounts can be read productively through Mishel’s theory of uncertainty in illness, which frames uncertainty as a pervasive cognitive state that arises when illness events are ambiguous, unpredictable, or informationally incomplete, and that patients then appraise as either a danger or an opportunity depending on the resources available to them (46). The narratives in this study repeatedly located the origin of distress not in the diagnosis itself but in the ambiguity that surrounded it: early reassurance followed by later malignancy, the four-month referral delay with associated stage progression, the chance discovery of metastatic status, the absence of pre-treatment preparation for chemotherapy side effects. In each case, uncertainty was not merely an emotional experience but a structural feature of the care pathway. Conversely, participants who described being carefully prepared for each treatment step appraised the same interventions as tolerable. This reading is consistent with recent work on metastatic cancer, where uncertainty and hope are found to co-exist in the era of open-ended targeted treatment (47), and it clarifies why the leverage points in our data consistently cluster around information, preparation, and continuity of contact rather than around mood per se.

The sociocultural context of Al-Ahsa shaped these accounts in ways that merit specific attention. First, faith functioned in the narratives less as a discrete coping strategy and more as an interpretive framework that re-organised the meaning of illness. Participant 6’s statement that she “leaves her affairs to God’s decree” and Participant 1’s invocation of the Qur’anic principle “God does not burden a soul beyond its capacity” were not adjuncts to treatment but templates for thinking about treatment. Second, the salience of marital structures within this cohort extends beyond presence or absence of a supportive spouse: Participant 9 described a polygamous marriage experienced as protective, while Participant 10 described a marriage in which abandonment compounded her metastatic-treatment crisis, and Participant 11’s divorced status was inseparable from her financial vulnerability. This suggests that “marital support” in this setting is a multi-dimensional construct that varies by family structure and cannot be captured by a single dichotomy. Third, several participants referenced preferences, implicit and explicit, for female clinicians and gender-appropriate care during intimate examinations, echoing findings in other Arab and Muslim-majority breast cancer populations and underlining the need for a workforce and facility design that accommodates these preferences (15).

Financial strain was a thread running across several accounts. Participant 1 described post-diagnosis employment loss and subsequent association-supported training; Participant 11 described metastatic disease compounded by divorce, two young children, and ongoing financial instability; and multiple participants described the Tafaoul Charitable Society’s role in bridging needs that fall outside direct clinical care. Although Saudi Arabia provides state-funded oncology care through the public system for eligible citizens, the out-of-clinic burden of illness, transport for treatment, caregiving gaps, lost work, and the costs of reconstruction or supplementary supportive care, was described as non-trivial, particularly for women without spousal income. These observations are consistent with a growing recognition that “financial toxicity” in breast cancer extends beyond direct medical costs and that charitable organisations fill a system-level gap whose implications for equitable survivorship deserve explicit study (48).

A key strength of the study is the depth of participant narratives, which reveal how clinical experiences interact with family responsibilities, social stigma, and system-level barriers to shape coping and wellbeing. The excerpts illustrate not only treatment burden but also the mechanisms through which psychological distress emerges, highlighting the role of information, pain control, and relational stability. The final sample of twelve participants, while modest in absolute terms, was judged to have adequate information power for an exploratory stage-sensitive analysis: the study had a narrow aim, a highly specific sample recruited through a single purposive channel, strong dialogue quality between interviewer and participant, and an established qualitative analytical strategy, all of which reduce the number of participants needed for analytic saturation (49). The large pool of invited but non-respondent women [approximately 300 invited, 20 expressing interest, 12 completing] nonetheless introduces a selection dimension that is itself informative: women who agreed to speak about illness experience may differ systematically from those who did not, and the findings should be read as illustrative of the experiences of women engaged with a charitable support organisation, rather than of all women with breast cancer in Al-Ahsa or Saudi Arabia. Analysis drew on the full interview transcripts rather than a pre-selected subset of excerpts, with reported quotations chosen to illustrate themes grounded in the complete dataset. The sample also reflects varied stages and circumstances, but may not capture the full diversity of experiences across regions, healthcare settings, or socio-economic backgrounds, limiting transferability. Translation from Arabic to English was performed by the lead author and cross-checked with the research team, with the English renderings of reported quotations independently checked by a second bilingual team member, but some loss of idiomatic, religious, or dialectal nuance is inherent in any qualitative translation process and should be acknowledged as a potential source of interpretive drift. Researcher positionality, the lead author is a male clinician working in Saudi Arabia, and the interviewer was a female member of the team, may have influenced what participants disclosed about intimate and marital experiences. To mitigate this, gender-sensitive topics were introduced by the female interviewer, and the team treated the male lead author’s clinical and cultural proximity to the setting as both an analytic resource and a potential source of bias, examining during analysis how it might have shaped coding and interpretation.

Findings suggest the need for strengthened early detection systems and improved referral coordination to reduce diagnostic delays and uncertainty. Clinically, structured patient education on treatment side effects, pain management, and realistic recovery expectations should be integrated early, alongside accessible communication channels with care coordinators. Routine psychosocial screening and proactive referral pathways may help identify high-risk situations, particularly where patients face severe symptoms, stigma, or marital instability. Future research could explore how support needs differ by stage, treatment type, and family context, and evaluate patient-navigation or psycho-oncology interventions aimed at improving both emotional outcomes and treatment adherence.

## 5 Conclusion

Within this single-site Al-Ahsa cohort of twelve women recruited through a charitable cancer support organisation, breast cancer experiences were shaped by an interplay of diagnosis timing, preparedness for treatment, symptom control, quality of clinician communication, marital and family context, and faith-based meaning-making, and these factors differed in emphasis across the stages of disease. When care was coordinated, pre-treatment information was specific, and support systems were protective, women described being able to endure treatment and maintain meaning-making; when referral delays, informational gaps, stigma, or relational rupture accumulated, distress escalated sharply and at times into safety-relevant crisis. These findings are exploratory and context-specific rather than generalisable, but they identify concrete and testable leverage points, timelier referral, structured stage-appropriate pre-treatment education, proactive and culturally sensitive psychosocial screening, and recognition of marital and charitable-support context in survivorship care, that warrant evaluation in larger, multi-site Saudi studies.

## Data Availability

The raw data supporting the conclusions of this article will be made available by the authors, without undue reservation.
