# Supplementary material for: Exploring the lived experiences of women with breast cancer: a qualitative study across different stages of the disease
Source: Front Public Health. 2026 Jul 1;14:1872393. doi: 10.3389/fpubh.2026.1872393 (PMC13368938; doi:10.3389/fpubh.2026.1872393)
Supplement: Supplementary file 1 [file Table_1.DOCX]

Supplementary Material

# Supplementary Data

Appendix A: Semi-Structured Interview Guide

Study aim: To explore women’s lived experiences of breast cancer diagnosis, treatment, and survivorship.

Interview format: Semi-structured, in-depth interviews

Language: Arabic

Estimated duration: 30–45 minutes

Setting: Private room / agreed safe location

Interviewer: Trained researcher with qualitative experience

Probing strategy: Follow-up prompts used as needed to encourage depth and clarification

A. Participant Background

1. Could you tell me a little about yourself?

o Age

o Marital status

o Level of education

o Number of children [if any]

2. Are you currently employed? If yes, what is your occupation?

3. How would you describe your daily activities or hobbies?

4. Do you have any other long-term or chronic medical conditions?

5. Is there a family history of breast cancer or other cancers?

B. Diagnosis Experience

6. Can you tell me about the time when you were first diagnosed with breast cancer?

7. How old were you at the time of diagnosis, and what stage was the cancer diagnosed at?

8. What prompted you to seek screening or medical evaluation?

9. How was the diagnosis communicated to you?

10. How did you feel when you received the diagnosis?

o Emotional reactions

o Initial thoughts or fears

11. What kind of information were you given at that time?

12. Did you find the diagnosis easy or difficult to understand? Why?

13. In your view, what do you think caused your illness?

o Do you believe stress or life pressures played a role?

o Did these factors affect your treatment experience?

14. Were you tested for genetic factors such as the BRCA gene?

15. If yes, were your family members informed or advised to undergo testing?

C. Treatment Journey

16. What types of treatment did you receive [e.g., surgery, chemotherapy, radiotherapy, hormone therapy]?

17. How long did your treatment last?

18. Did you undergo a unilateral or total mastectomy?

19. Were you offered prophylactic mastectomy? How did you feel about that option?

20. Were there any treatments you declined or hesitated to accept?

o What influenced that decision?

21. Did you receive hormone therapy?

o Did you have any concerns or fears regarding it?

22. How did the treatment affect you physically?

23. What side effects did you experience, and how did you manage them?

24. How did you feel emotionally and psychologically during treatment?

25. Were you involved in making decisions about your treatment?

o How did that level of involvement make you feel?

26. Did you use any complementary or alternative therapies?

27. What are your thoughts about alternative treatments you have heard about?

28. What is your opinion on dietary approaches, such as reducing sugar intake, to limit cancer progression?

29. Were you offered reconstructive or cosmetic surgery?

o What were your thoughts or concerns regarding this option?

30. Were you offered psychological support or referred for counseling?

31. Were you offered fertility preservation options such as egg freezing?

o What are your views on this?

D. Psychological and Emotional Impact

32. How did the diagnosis and treatment affect your mental health overall?

33. Did you experience anxiety, depression, or fear?

34. How did you cope with these feelings?

35. Were there any family-related challenges before or after treatment?

36. Did you receive emotional or psychological support from family, friends, or professionals?

E. Social Support and Relationships

37. How has breast cancer affected your relationships with:

• Family members

• Friends

• Colleagues or workplace relationships?

38. Did you feel supported by your community?

39. How would you describe your experiences with healthcare providers in terms of support and communication?

F. Quality of Life

40. How has your quality of life changed since your diagnosis?

41. Were there changes in your daily routine, work, or leisure activities?

42. Do you feel your life has improved, worsened, or returned to what it was before diagnosis?

43. What strategies or practices helped you maintain or improve your quality of life?

G. Survivorship and Long-Term Concerns

44. Are you currently undergoing treatment, or are you in remission?

45. How do you feel about the future?

46. What are your main concerns related to breast cancer at this stage of your life?

47. Do you attend regular follow-up appointments?

48. What types of follow-up tests or investigations are you currently undergoing?

H. Recommendations and Reflections

49. What advice would you give to women who have recently been diagnosed with breast cancer?

50. In your opinion, what aspects of breast cancer care could be improved?

51. Would you advocate for changes in diagnosis, treatment, or support services?

52. Would you recommend early breast cancer screening? Why or why not?

Closing Question

53. Is there anything else you would like to share about your experience that we have not discussed?Supplementary material is not typeset so please ensure that all information is clearly presented, the appropriate caption is included in the file and not in the manuscript, and that the style conforms to the rest of the article.

# Supplementary Data

Appendix B: COREQ checklist

54. Consolidated criteria for reporting qualitative studies [COREQ]: 32-item checklist

| Item No | Guide Questions/Description | Reported on Page # |  |  |
| --- | --- | --- | --- | --- |
| Domain 1: Research team and reflexivity | | |  |  |
| Personal Characteristics | | |  |  |
| 1. Interviewer/ facilitator | Which author/s conducted the interview or focus group? | Pg 3 |  |  |
| 2. Credentials | What were the researcher’s credentials? E.g., PhD, MD | Pg 1 |  |  |
| 3. Occupation | What was their occupation at the time of the study? | Pg 1 |  |  |
| 4. Gender | Was the researcher male or female? | Pg 1 |  |  |
| 5. Experience and training | What experience or training did the researcher have? | Pg 1 |  |  |
| Relationship with participants | | |  |  |
| 6. Relationship established | Was a relationship established prior to study commencement? | Pg 3 |  |  |
| 7. Participant knowledge of the interviewer | What did the participants know about the researcher? e.g. personal goals, reasons for doing the research? | Pg 2 |  |  |
| 8. Interviewer characteristics | What characteristics were reported about the interviewer/facilitator? e.g. Bias, assumptions, reasons and interests in the research topic | Pg 4 |  |  |
| Domain 2: study design | | |  |  |
| Theoretical framework | | |  |  |
| 9. Methodological orientation and Theory | What methodological orientation was stated to underpin the study? e.g. grounded theory, discourse analysis, ethnography, phenomenology, content analysis | Pg 4 |  |  |
| Participant selection | | |  |  |
| 10. Sampling | How were participants selected? e.g., purposive, convenience, consecutive, snowball | Pg 3 |  |  |
| 11. Method of approach | How were participants approached? e.g., face-to-face, telephone, mail, email | Pg 3 |  |  |
| 12. Sample size | How many participants were in the study? | Pg 3 |  |  |
| 13. Non-participation Setting | How many people refused to participate or dropped out? Reasons? | Pg 3 |  |  |
| 14. Setting of data collection | Where was the data collected? e.g., home, clinic, workplace | Pg 4 |  |  |
| 15. Presence of nonparticipants | Was anyone else present besides the participants and researchers? | N/A |  |  |
| 16. Description of sample | What are the important characteristics of the sample? e.g. demographic data, date | Pg 3 |  |  |
| Data collection | | |  |  |
| 17. Interview guide | Were questions, prompts, and guides provided by the authors? Was it pilot tested? | Pg 4 |  |  |
| 18. Repeat interviews | Were repeat interviews carried out? If yes, how many? | N/A |  |  |
| 19. Audio/visual recording | Did the research use audio or visual recording to collect the data? | Pg 3 |  |  |
| 20. Field notes | Were field notes made during and/or after the interview or focus group? | Pg.4 |  |  |
| 21. Duration | What was the duration of the interviews or focus group? | Pg 4 |  |  |
| 22. Data saturation | Was data saturation discussed? | Pg 3 |  |  |
| 23. Transcripts returned | Were transcripts returned to participants for comment and/or correction? | N/A |  |  |
| Domain 3: analysis and findings | | |  |  |
| Data analysis | | |  |  |
| 24. Number of data coders | How many data coders coded the data? | Pg 4 |  |  |
| 25. Description of the coding tree | Did the authors provide a description of the coding tree? | N/A |  |  |
| 26. Derivation of themes | Were themes identified in advance or derived from the data? | Pg 5 |  |  |
| 27. Software | What software, if applicable, was used to manage the data? | Pg 5 |  |  |
